# MED18/388: Defining the Optimal Framework Required for the Development of Multimedia Web-based Training: The Mater Misericordiae Hospital / Institute of Healthcare Informatics experience

**DOI:** 10.2196/jmir.1.suppl1.e61

**Published:** 1999-09-19

**Authors:** B Moran

**Affiliations:** ^1^Mater Misericordiae Hospital, DublinIreland

**Keywords:** Web-Based Training, Education, Multimedia Tool-Sets, Distance Learning, Internet

## Abstract

**Introduction:**

The provision of education, training and pedagogy, its associated science is being radically impacted by technological developments and changing methodologies in the imparting and acquisition of knowledge. This is resulting in the thrust and focus of higher education moving from a traditional university centred process to a more student-orientated one. The Internet's propensity for real-time interaction makes computer-based training, or distance learning, viable for accommodating medical education needs. In medicine many concepts are visual or structural and in many cases clinical reasoning is based on spatial abstraction of structures. Incorporating these principles, the Institute developed the concept of the Virtual Study Centre (VSC), utilising methodologies which take advantage of the developments in web based computing and communication infrastructures to specifically address the difficulties associated with existing methods of distance learning.

**Methods:**

The VSC is a 'hypermedia' learning environment, which offers a more flexible approach to learning for both producers and users of educational material. It is achieved through distributed student access to networked learning resources. A project team was established to define the specific models of learning scenarios where synchronous interaction played an important role. Utilising an assessment matrix based on the requirement outcomes of the learning scenarios, it evaluated products available in the web-based training area for quality, extent and complexity in respect to multimedia design, interactivity and overall ease of use. Each product had to comply with their criteria under the following headings: technical composition, infra-structural design, regional and cultural constraints. A compromise in the quality and speed of audio and video was not an option. To provide the best we expected the best from the products so that we could provide a well-crafted teaching system, which could be used throughout the medical profession.

**Results:**

We have identified the optimal model based standards required in software tool-sets to develop and support multimedia web-based training.

**Discussion:**

A fundamental element in the provision of distance education is interaction. This paper illustrates the considerable deficit in the availability of quality media-rich tool-sets on the market, which enables optimum interaction. It details the research undertaken in the provision of the levels of interaction necessary to provide a fully integrated web-based education facility and the difficulties of tailoring a traditionally taught course for the web. It develops and explores the issues surrounding the monitoring, examination and identification of students and defines an approach to underpin optimum tool-set assessment and utilisation.

